# Aromatic Amine-Functionalized Covalent Organic Frameworks (COFs) for CO_2_/N_2_ Separation

**DOI:** 10.1021/acsami.2c17672

**Published:** 2023-01-17

**Authors:** Ellen Dautzenberg, Guanna Li, Louis C.P.M. de Smet

**Affiliations:** † Laboratory of Organic Chemistry, Wageningen University and Research, Stippeneng 4, 6708WEWageningen, The Netherlands; ‡ Biobased Chemistry and Technology, Wageningen University and Research, Bornse Weilanden 9, 6708WGWageningen, The Netherlands

**Keywords:** covalent organic frameworks, dynamic covalent chemistry, linker exchange, gas separation, carbon capture

## Abstract

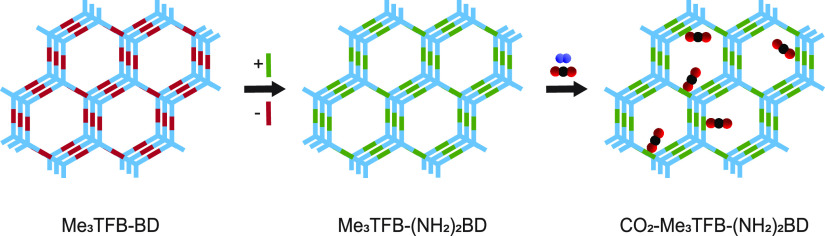

CO_2_ is a prominent example for an exhaust gas, and it is known for its high impact on global warming. Therefore, carbon capture from CO_2_ emissions of industrial processes is increasingly important to halt and prevent the disruptive consequences of global warming. Covalent organic frameworks (COFs) as porous nanomaterials have been shown to selectively adsorb CO_2_ in high quantities and with high CO_2_/N_2_ selectivity. Interactions with amines are recognized to selectively adsorb CO_2_ and help capture it from exhaust emissions. Herein, a novel COF (Me_3_TFB-(NH_2_)_2_BD), which was not accessible via a direct condensation reaction, was synthetized by dynamic linker exchange starting with Me_3_TFB-BD. Despite the linker exchange, the porosity of the COF was largely maintained, resulting in a high BET surface area of 1624 ± 89 m^2^/g. The CO_2_ and N_2_ adsorption isotherms at 273 and 295 K were studied to determine the performance in carbon capture at flue gas conditions. Me_3_TFB-(NH_2_)_2_BD adsorbs 1.12 ± 0.26 and 0.72 ± 0.07 mmol/g of CO_2_ at 1 bar and 273 and 295 K, respectively. The COF shows a high CO_2_/N_2_ IAST selectivity under flue gas conditions (273 K:83 ± 11, 295 K: 47 ± 11). The interaction of the aromatic amine groups with CO_2_ is based on physisorption, which is expected to make the regeneration of the material energy efficient.

## Introduction

Porous materials are a class of solid, permanently porous materials with pore sizes of a few to several hundred nanometers. These pores can be classified into macropores (>50 nm), mesopores (2–50 nm), and micropores (<2 nm).^1^ In 2005, Yaghi and co-workers^2^ reported the first covalent organic framework (COF) as a new type of fully organic porous material. Since then, COFs have gained increasingly more interest and a huge variety of different materials and applications have been reported.^3−5^ Their permanent porosity and channel-like structure lead to a high surface area, which is of interest for, among others, energy storage,^3,6^ chemical sensing,^3,7^ photocatalysis,^3,8,9^ and gas separation.^3,10^ Usually, two multifunctional building blocks are linked by dynamic covalent chemistry (DCC).^11,12^ DCC refers to reversible reactions carried out under thermodynamic reaction conditions, which enables error correction within the reaction, leading to crystalline frameworks with long-range order. Additionally, the exchange of building blocks (linker exchange) in already existing, crystalline frameworks opens the possibility to synthesize COFs with improved crystallinity^13^ or make COFs accessible that were not accessible via condensation of the respective building blocks.^14,15^ Qian et al. reported the first COF-to-COF linker exchange by replacing 1,4-phenylenediamine (PA) with benzidine (BD) in an imine-linked COF, which yielded an almost complete transformation.^16^ The linker-exchange strategy was applied to achieve, among others, different COF linkages,^14,17−20^ changes in the pore hierarchy and size by different linker symmetries,^21−23^ the transformation of linear polymers into COFs^24,25^ or 3D into 2D COFs,^26^ and the introduction of functional groups into the framework.^27^

Being a primary greenhouse gas, CO_2_ plays an important role in climate change. Strategies to mitigate global warming included the reduction of CO_2_ emissions via carbon capture and storage. The separation of CO_2_ from N_2_—and the associated CO_2_/N_2_ selectivity—play a crucial role in such strategies. In more detail, CO_2_ capture is industrially performed by amine solutions that react with it.^28,29^ However, in this case the CO_2_ covalently binds, which means that the regeneration process requires a lot of energy. Alternatively, porous materials as outlined above are promising candidates for carbon capture because their large surface area enables them to store large quantities of CO_2_. To store CO_2_, it must first be—ideally selectively—separated from a gas mixture, for example, from the atmosphere or flue gas. For direct air capture, 400 ppm of CO_2_ in N_2_ is relevant because this is the current atmospheric CO_2_ concentration.^30,31^ Coal flue gas contains 10–25 vol % CO_2_.^32^ Capturing the CO_2_ and separating it from the other gases before the flue gas gets diluted by the atmosphere could help to reduce CO_2_ emissions into the atmosphere.^30,31^ In this context, the porosity of COFs is advantageous to store large quantities of gas.^33,34^

The CO_2_/N_2_ selectivity can be calculated out of pure component isotherms based on the ideal adsorption solution theory (IAST), which was developed by Myers and Prausnitz.^35^ To simulate the adsorption behavior of gas mixtures, the pure component isotherms must be measured at the same temperature and on the same adsorbent. The basis of IAST is analogous to Raoult’s law for vapor–liquid equilibrium.^35^ For IAST, the adsorbed phase is assumed to behave like an ideal solution that is in an equilibrium with the bulk adsorptive. It is assumed that the adsorbent is thermodynamically inert, that the Gibbs definition of adsorption applies, and that the surface area is universally accessible and temperature-invariant. It must be noted that IAST is only of limited validity for polar adsorptives or mixtures, in which one component strongly adsorbs and the other one weakly. Other limitations are heterogeneous adsorbents and low loadings as they can lead to poor predictions of mixture adsorption. In more detail, for example, low loadings can lead to inaccurate spreading pressures, falsifying the selectivity calculation. If the fitted isotherms do not accurately reproduce the Henry constant for the specific adsorbate, the IAST calculation will predict incorrect selectivity values.^36^ Nevertheless, the simplicity of these calculations makes IAST a widely used method for selectivity determination and provides good results for screening the applicability of porous materials in gas separation. The selectivity *S* of a binary gas mixture in IAST is defined as the ratio of the mole fractions in the adsorbed state (*q*) over the mole fractions of the bulk phase (*p*) of components 1 and 2 (eq 1).^37^
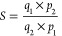
1

Several different porous materials have been investigated for such CO_2_/N_2_ separations under flue gas conditions, among others benzimidazole-linked porous organic polymers (POPs). They have been shown to store CO_2_ in large quantities (up to 5.19 mmol/g at 273 K and 1 bar) with good CO_2_/N_2_ selectivity values (63 at 298 K and 1 bar in a 0.1/0.9 mixture).^38−45^ Post-synthetic functionalization can further enhance the selectivity.^41^ The well-known amine–CO_2_ interaction^28,29^ makes the amine-functionalized benzidine linker 3,3′-diaminobenzidine ((NH_2_)_2_BD) interesting for CO_2_/N_2_ separation. For example, this linker was employed to synthesize a benzimidazole-linked COF (IISERP-COF3),^46^ a semi-crystalline covalent organic polymer,^47^ and benzimidazole POPs for CO_2_ adsorption.^42,43^ Besides benzimidazole-linked porous materials, imine-linked materials have also been investigated, which often have CO_2_/N_2_ IAST selectivity values in the range of 10–30 without additional pore–wall functionalization that would then be present inside the pores.^48,49^ To the best of our knowledge, the highest CO_2_/N_2_ IAST selectivity value so far (185.8 at 273 K for 15% CO_2_) was reported by Mahato et al. for a covalent triazine framework.^50^ Recently, Yaghi and co-workers studied the incorporation of aliphatic amine groups into a tetrahydroquinoline COF.^51^ They found high CO_2_ adsorptions of 0.304 mmol/g at 0.4 mbar and 25 °C, which are relevant conditions for direct air capture, and that 50% humidity even enhances the CO_2_ uptake to 0.393 mmol/g at 298 K and 1 bar. Based on solid-state NMR, they showed that carbamates were formed during the adsorption, therefore confirming chemisorption interactions.

Thus, functional groups are important for the interaction of porous materials with CO_2_ to achieve high adsorption quantities and high selectivity values for carbon capture. The nitrogen-CO_2_ interaction—especially the one of amine-CO_2_—has shown to be beneficial in terms of adsorption and selectivity and can be used for the rational design of novel materials for carbon capture. However, the chemical properties, e.g., the p*K*_a_ value, are dependent on the nature of the nitrogen atom. Aliphatic amines, as used by Yaghi and co-workers,^51^ can interact more strongly with CO_2_ compared to aromatic amines, leading to chemisorption. Since chemisorption requires higher energies for the regeneration of the material than physisorption, we decided to use aromatic amines, which are directly linked to the COF backbone. In a classical condensation reaction, these additional amine groups are expected to interfere with the framework formation, so we used the dynamic linker-exchange strategy to obtain Me_3_TFB-(NH_2_)_2_BD). After the characterization of the novel COF, we further studied the CO_2_/N_2_ adsorption and selectivity under flue gas conditions for gas separation.

## Experimental Section

### Materials

3,3′-Diaminobenzidine (>98%, HPLC) was purchased from TCI Europe N.V., benzidine (98%) was purchased form Abcr, and all chemicals were used without further purification. Mesitylene (99%, extra pure) was purchased from Fisher Scientific, 1,4-dioxane (99%) was purchased from Acros Organics B.V.B.A., and 2,5,6-trimethyl-1,3,5-benzenetricarboxaldehyde was synthesized before.^52^ All other solvents, lab supplies, and glacial acetic acid (AR) were purchased from commercial sources and used without further purifications.

### Instrumentation

^1^H and ^13^C{^1^H} cross-polarization magic angle spinning (CPMAS) solid-state NMR (ssNMR) spectra were recorded on a Bruker AVANCE III HD spectrometer at 700.13 MHz (16.4 T) and 176 MHz, respectively. Solid-state NMR samples were packed into 4 mm zirconia rotors and spun at MAS frequencies of 11 and 14 kHz at 298 K. The ^13^C CPMAS spectra were recorded by using a CP pulse sequence. SPINAL64 decoupling was applied on protons with a decoupling strength of 104 kHz. The ^13^C CPMAS spectra were obtained with a recycle delay of 3 s, and the strength of the CP contact pulses of 0.08 kHz related to a contact time of 3 ms unless stated differently. The ^13^C ssNMR spectra were referenced with respect to adamantane (^13^C, 29.456 ppm). The spectra were analyzed using MestReNova (version 14.1.0).

FT-IR spectra were obtained on a Bruker Tensor 27 spectrometer with an attenuated total reflection accessory called Platinum. The samples were applied as powder on top of the crystal. A total of 64 scans were performed with a resolution of 4 cm^–1^.

Powder X-ray diffraction measurements were recorded with a Philips X’pert-PRO at 40 kV and 40 mA from 4–40° (step size: 0.05°, step time: 90 s, mask in front of entrance: 10 mm and slid 1°, slid before detector: 1°) and from 1.5–10° (step size: 0.05°, step time: 500 s, mask in front of entrance: 5 mm and slid 0.5°, slid before detector: 0.25°). X-rays were generated by a Cu anode Kα (1.54 λ) radiation.

Nitrogen adsorption–desorption measurements were performed on a MicroActive for Tristar II Plus 3030 at 77.350 K. Before the measurement, the samples were outgassed at 120 °C overnight. Surface areas were calculated from the adsorption data using Brunauer–Emmet–Teller (BET) methods and Rouquerol criteria. The fitting parameters are 2.643 ± 0.142 and 4.29 ± 0.16 × 10^–5^ for the slope and intercept, respectively. The pore-size distribution curves were obtained from the adsorption branches using the method “HS-2D-NLDFT, Carb Cyl Pores (ZTC) N2@77K”. An optimum between Goodness of Fit and smoothness of the pore size distribution was aimed for. The average of three different COF batches was used to determine the BET surface areas. Nitrogen and carbon dioxide adsorption measurements were performed at 295 K with absolute pressure dosing and an equilibration time of 20 s between 3 and 1200 mbar. The free space values were determined after the adsorption measurement. Nitrogen measurements at 273 K were performed under the same conditions as those at 295 K. Carbon dioxide measurements at 273 K were performed by increment dosing up to *p*/*p*^0^ = 0.03 with an increment of 0.13384 mmol/g and an equilibration time of 20 s. The gases were considered as ideal gases under all these circumstances. Isotherm cycling was performed without a degassing step in between. At 273 K, the adsorption branch was measured up to *p*/*p*^0^ = 0.03 with an increment of 0.15 mmol/g, and the desorption branch was measured down to *p*/*p*^0^ = 0.0003 with an increment of 0.15 mmol/g. At 295 K, the adsorption branch was measured between 3 and 1050 mbar, and the desorption branch was measured between 1050 and 12.5 mbar.

The CO_2_/N_2_ selectivities were predicted based on the ideal adsorption solution theory (IAST)^35,36^ by GraphIAST^53^ at 1 bar and a composition of 0.15/0.85. The isotherms were fitted with the interpolator model.

Thermogravimetric analysis was performed on a PerkinElmer STA 6000. The sample was heated to 30 °C, the temperature was held for 1 min, and afterward, the sample was heated with 10.00 °C/min to 700 °C in a nitrogen atmosphere (20 mL/min).

### Computational Details

All DFT calculations for COF structures were performed by using the Vienna Ab Initio Simulation Package (VASP, version 5.4.4).^54,55^ The PBE functional based on the generalized gradient approximation was chosen to account for the exchange–correlation energy.^56^ A plane-wave basis set in combination with the projected augmented wave (PAW) method was used to describe the valence electrons and the valence–core interactions, respectively.^57^ The kinetic energy cut-off of the plane wave basis set was set to 500 eV. Gaussian smearing of the population of partial occupancies with a width of 0.05 eV was used during iterative diagonalization of the Kohn–Sham Hamiltonian. The threshold for energy convergence for each iteration was set to 10^–5^ eV. Geometries were assumed to be converged when forces on each atom were less than 0.05 eV/Å. The Brillouin zone integration and *k*-point sampling were done with a Gamma-centered 1 × 1 × 8 and 2 × 2 × 4 grid points for the eclipsed and staggered unit cells, respectively. The Van der Waals (vdW) interactions were included by using Grimme’s DFT-D3(BJ) method as implemented in VASP.^58^ Simulated XRD patterns were obtained by using VESTA (version 3.4.8).^59^ Coordinates of all crystal structures are provided as separate files.

### Synthesis of Me_3_TFB-(NH_2_)_2_BD

Me_3_TFB-COF (100.7 mg, 0.236 mmol, 1 equiv) and (NH_2_)_2_BD (75.8 mg, 0.354 mmol, 1.5 equiv) were added to a 50 mL round-bottom flask (RBF), together with a stirring rod, and were dissolved in 10 mL of 4:1 v/v 1,4-dioxane:mesitylene mixture. The mixture was heated to 70 °C for 5 min. Water (0.42 mL) and glacial acetic acid (0.56 mL) were added. The reaction mixture was stirred at 70 °C for 3 days. Afterward, the reaction was cooled to RT and the precipitate was collected via Büchner filtration. The solid was dispersed in dimethylformamide (DMF), stirred at 90 °C for 30 min, and collected via Büchner filtration. These steps were repeated with DMF (90 °C, 30 min), ethanol (80 °C, 30 min), acetone (60 °C, 30 min), and hexane (70 °C, 30 min). After the final Büchner filtration, the COFs were dried overnight at 120 °C in a regular oven. Brown powder (86.3 mg, 0.183 mmol, 78%) was obtained. After drying, the COFs were kept in a glovebox for storage.

The synthesis was repeated in total three times: twice with the amounts specified and once scaled-up to 200 mg of Me_3_TFB-COF.

The direct condensation of Me_3_TFB-(NH_2_)_2_BD was carried out according to our previously published synthesis procedure.^52^

## Results and Discussion

In an attempt to directly synthesize the amine-functionalized COF, 2,4,6-trimethylbenzene-1,3,5-tricarbaldehyde (Me_3_TFB) and 3,3′-diaminobenzidine ((NH_2_)_2_BD) were reacted in a 1:4 v/v mixture of mesitylene:1,4-dioxane. Acetic acid was added as a catalyst for the reaction. The reaction mixture was heated to 70 °C for 3 days before the obtained precipitate was isolated by Büchner filtration and subjected to the extensive washing procedure from Dichtel and co-workers.^60^ Afterward, the obtained powder was dried at 120 °C overnight in a regular oven before the characterization was carried out. The results of Fourier-transform IR spectroscopy (FT-IR), powder X-ray diffraction (PXRD) and N_2_ sorption analysis indicate the formation of an amorphous polymer without crystallinity and porosity (Figures S1–S3).

**Scheme 1:**
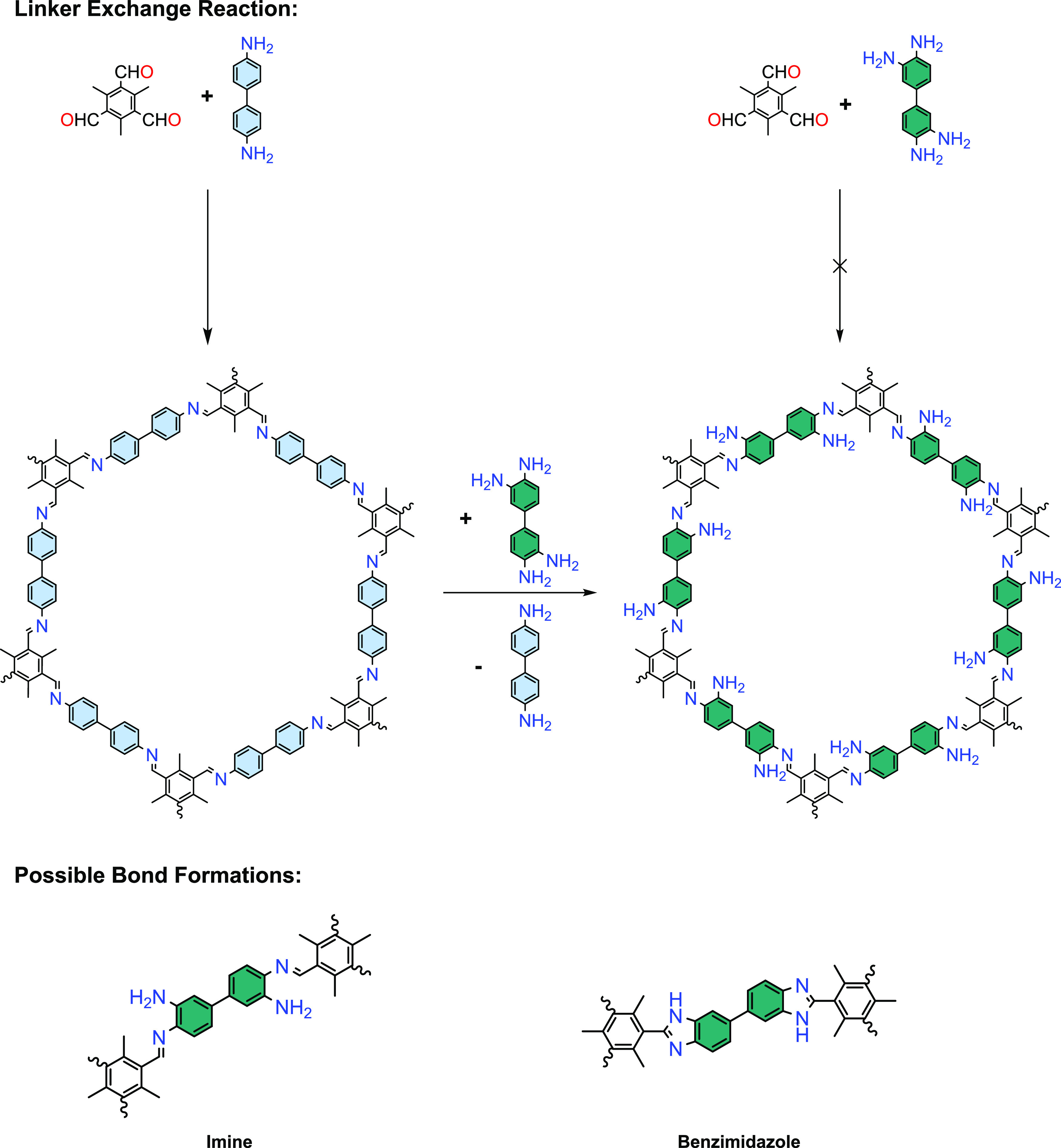
Schematic Overview of the Me_3_TFB-BD COF Reaction and the Following Linker-Exchange with (NH_2_)_2_BD to Me_3_TFB-(NH_2_)_2_BD (Top) The direct condensation from the building blocks failed (top right). Here, the idealized structures are depicted. In theory two different bonds could be formed: imine bonds with primary amine substituents (bottom left) or benzimidazole bonds (bottom right).

Then, the known Me_3_TFB-BD COF was synthesized by condensation of Me_3_TFB and benzidine (BD) according to a previously published procedure.^52^ Afterward, Me_3_TFB-BD was dispersed in a 1:4 v/v mixture of mesitylene:1,4-dioxane together with acetic acid and water to achieve an equilibrium between formed and broken imine-bonds. Additionally, (NH_2_)_2_BD was added, which can exchange with benzidine (Scheme 1). The reaction was carried out at 70 °C for 3 days before the same work-up procedure as before was applied. Afterward, the obtained powder was again dried at 120 °C overnight in a regular oven. Upon the linker-exchange reaction, the color of the COF changed from light yellow to brown. The COF synthesis was repeated two more times to achieve independent triplicate analysis during characterization of the material’s properties. The COF synthesis was carried out at a 400 mg (2 mmol) scale of Me_3_TFB, and the linker exchange was carried out with up to 200 mg of pristine COF.

Theoretically, the linker-exchange reaction can lead to two different bond formations, namely, imine bonds with unreacted primary amines as *ortho*-substituents or benzimidazole bonds (Scheme 1). Both bond formations are reported for porous materials.^38,46^ Here, we thoroughly characterized the COF by solid-state NMR and FT-IR to determine which bond(s) has/have been formed.

**Figure 1:**
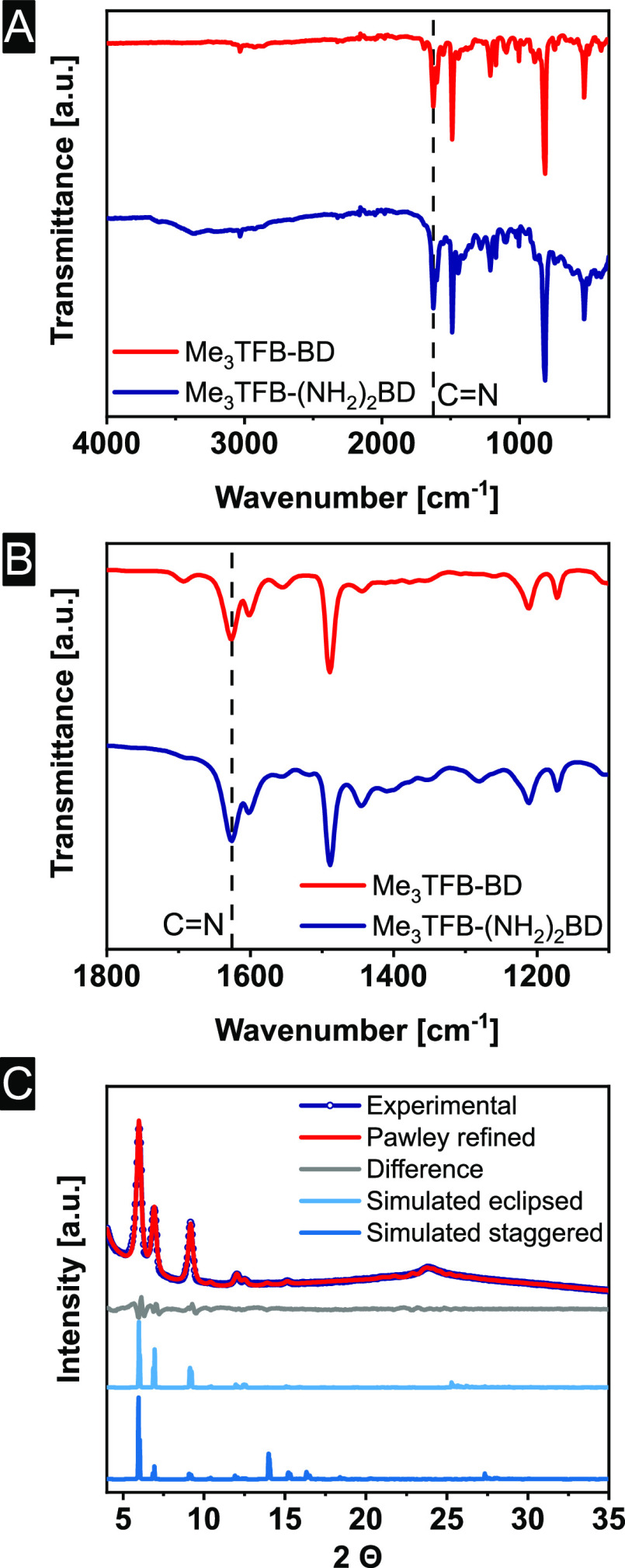
(A) FT-IR spectra of Me_3_TFB-(NH_2_)_2_BD and Me_3_TFB-BD. The C=N stretching band at 1626 cm^–1^ and the N–H stretching band between 3600 and 3000 cm^–1^ indicate the formation of imine bonds and primary amines. (B) Zoom-in into the relevant wavenumbers for C=O and C=N stretches. No leftover C=O stretching bands are visible, indicating the complete conversion of aldehydes into imine bonds. (C) PXRD pattern of Me_3_TFB-(NH_2_)_2_BD, including Pawley refinement, the difference between the experimental and Pawley-refined pattern, and simulated eclipsed and staggered PXRD patterns.

To study the linker exchange, the FT-IR spectrum of Me_3_TFB-(NH_2_)_2_BD was measured and compared to the one of Me_3_TFB-BD. In short, the C=N imine band at 1627 cm^–1^ indicates the formation of imine bonds (Figure 1A,B and Figure S4). A broad band is visible between 3600 and 3000 cm^–1^, which can be assigned to the N–H stretch, confirming the linker exchange. The band at 1693 cm^–1^ that can be assigned to C=O stretching is significantly smaller, indicating a low amount of aldehyde groups likely present only at the periphery of the 2D sheets.^61−63^ However, FT-IR cannot be used to unambiguously assign the bond formation because the respective bands (imine: 1627 cm^–1^, benzimidazole: 1610–1625 cm^–1^)^38,45,52^ are too close to each other.

The PXRD patterns of Me_3_TFB-(NH_2_)_2_BD display several diffraction peaks, which are clearly baseline-separated from each other at 3.5, 6.0, 6.9, 9.2, 12.0, 12.5, and 15.2°, and a broad peak at 24° (Figure 1C). The peak at 3.5° could only be detected with a special low-angle measurement (Figure S8). Pawley refinement was performed in triplicate (Figure S6,7), using the space group *P*6*/m*, which corresponds to a hexagonal, eclipsed stacking structure. The refined unit cell dimensions are *a* = 29.58 ± 0.05 Å and *c* = 3.75 ± 0.08 Å with *R_wp_* = 3.44 ± 0.46% and *R_p_* = 2.31 ± 0.26%. The unit cell dimensions are close to those of Me_3_TFB-BD COF.

To obtain insight in the stacking of the COF sheets, the structures of two extreme models referred to as “eclipsed” and “staggered” were optimized using DFT. The DFT optimization was performed by using the Vienna Ab Initio Simulation Package (VASP); further details can be found in the Supporting Information. Next, the PXRD diffractograms were modeled for both computed structures. The simulated PXRD diffractions of the eclipsed pattern (Figure S24) match well with the experimental PXRD result, indicating an eclipsed stacking conformation for Me_3_TFB-(NH_2_)_2_BD (Figure 1C). The deviation between the computed unit cell dimensions and the Pawley-refined values is mostly below 5% except for the interlayer distance *c*, which deviates by 6.5%. This is within the expected accuracy of the DFT calculations, further supporting the formation of eclipsed COFs.^64^

These PXRD results confirm the formation of a crystalline Me_3_TFB-(NH_2_)_2_BD COF, which could not be obtained via the classical one-step condensation reaction. Thereby, the linker-exchange strategy provides a powerful tool to obtain previously inaccessible COFs with high crystallinity even if the functional group on the linker molecule can interfere with the bond formation.

**Figure 2:**
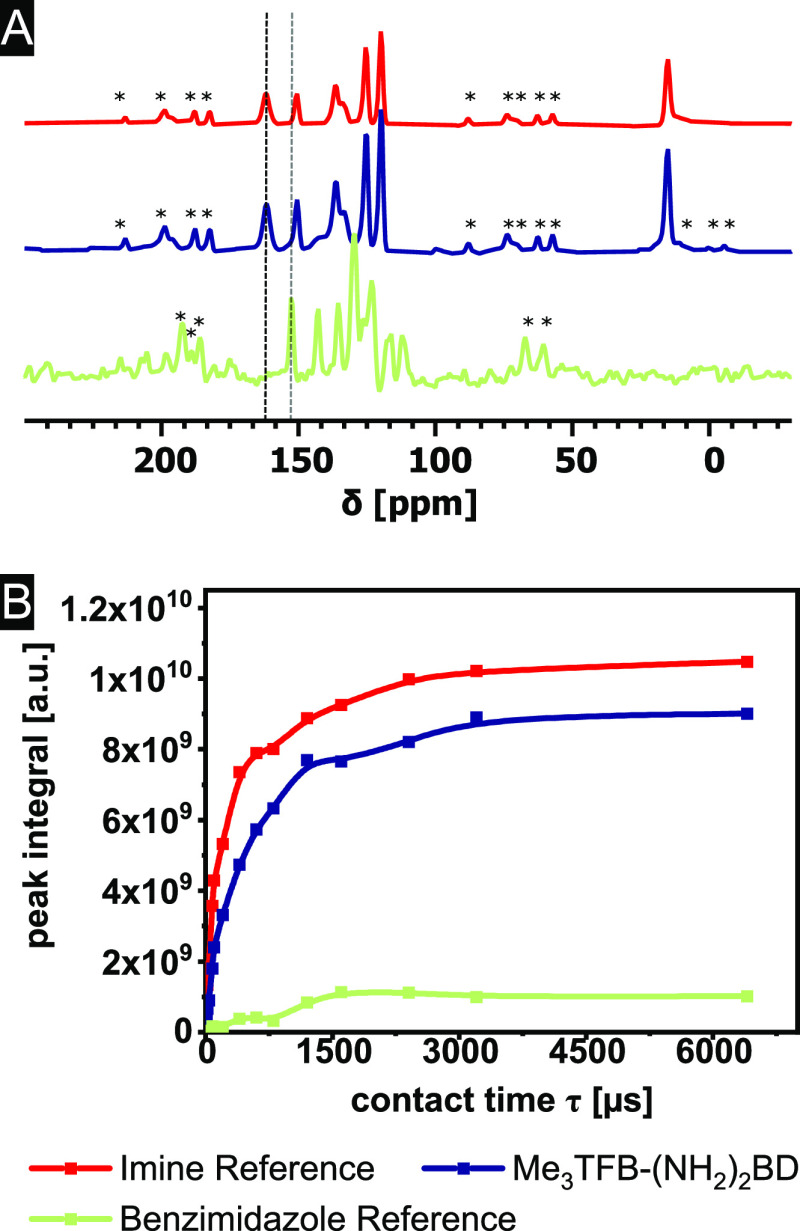
(A) ^**13**^C CPMAS ssNMR spectra of Me_3_TFB-BD (red) as a reference imine COF, 2-phenylbenzimidazole (green) as benzimidazole model compound, and Me_3_TFB-(NH_2_)_2_BD (blue) at 11 kHz spinning frequency at 3.2 ms CP contact time. (B) CP build-up curves of the respective samples. Spinning side bands are denoted with an asterisk.

To unambiguously assign the linkage, ^13^C cross-polarization magic angle spinning solid-state NMR (^13^C CPMAS ssNMR) was conducted. The spectrum of the newly obtained Me_3_TFB-(NH_2_)_2_BD sample as well as its CP build-up curve were measured (Figures S10–S12). In comparison with spectra of the small model compound 2-phenylbenzimidazole (Figures S13–S15) and Me_3_TFB-BD,^65^ the linkage was found to consist of imine bonds (Figure 2). In more detail, the chemical shift of the benzimidazole carbon is at 152 ppm and the shift of the imine carbon at 162 ppm (Figure 2A). The ssNMR spectrum of Me_3_TFB-(NH_2_)_2_BD shows a signal at 162 ppm, clearly indicating imine-bond formation. Additionally, the CP build-up curve of the imine COF (Me_3_TFB-BD) matches with the CP build-up curve of Me_3_TFB-(NH_2_)_2_BD, while 2-phenylbenzimidazole shows a significant slower build-up (Figure 2B). This was expected because a benzimidazole carbon atom is not directly bound to a proton, slowing the CP build-up down. Compared to the ssNMR spectrum of Me_3_TFB-BD, the additional signal at 142 ppm appears due to the amine substituent on the benzidine, which shifts the signal downfield. The presence of the signals at 142 ppm (*C*-NH_2_) as well as 120 ppm (*C*-H) indicates the formation of a partly exchanged material.

Digestion ^1^H-NMR was attempted, but the COF was too stable to be completely broken down to monomers. In NMR digestion, it is aimed to depolymerize a material. Here, the COF was attempted to depolymerize into its monomers by using DCl in D_2_O in deuterated DMSO-*d*_6_, before analyzing the resulting solution by ^1^H-NMR. Compared to Me_3_TFB-BD, which could be digested under the same conditions,^66^ no clear solution of digested Me_3_TFB-(NH_2_)_2_BD could be obtained, but a dispersion was obtained. Thus, the post-synthetic linker exchange seems to enhance the chemical stability in acidic media. To further support this, Me_3_TFB-(NH_2_)_2_BD was immersed in 1 M of HCl for 5 days, and upon re-isolation and drying at 120 °C overnight, the PXRD pattern was recorded again. The COF could be isolated from the vial used for the stability test, showing that the material was not completely amorphized. The disappearance of the PXRD diffractions indicates that the crystallinity was not retained (Figure S9). Therefore, Me_3_TFB-(NH_2_)_2_BD shows an enhanced acid stability over Me_3_TFB-BD but cannot yet be considered as stable under these conditions.

The thermal stability was investigated using thermogravimetric analysis (TGA). The threshold for thermal stability was determined at the position where the COF lost maximum 5% of its initial weight. Me_3_TFB-(NH_2_)_2_BD COF shows an excellent thermal stability up to 438 °C (Figure S23).

**Figure 3:**
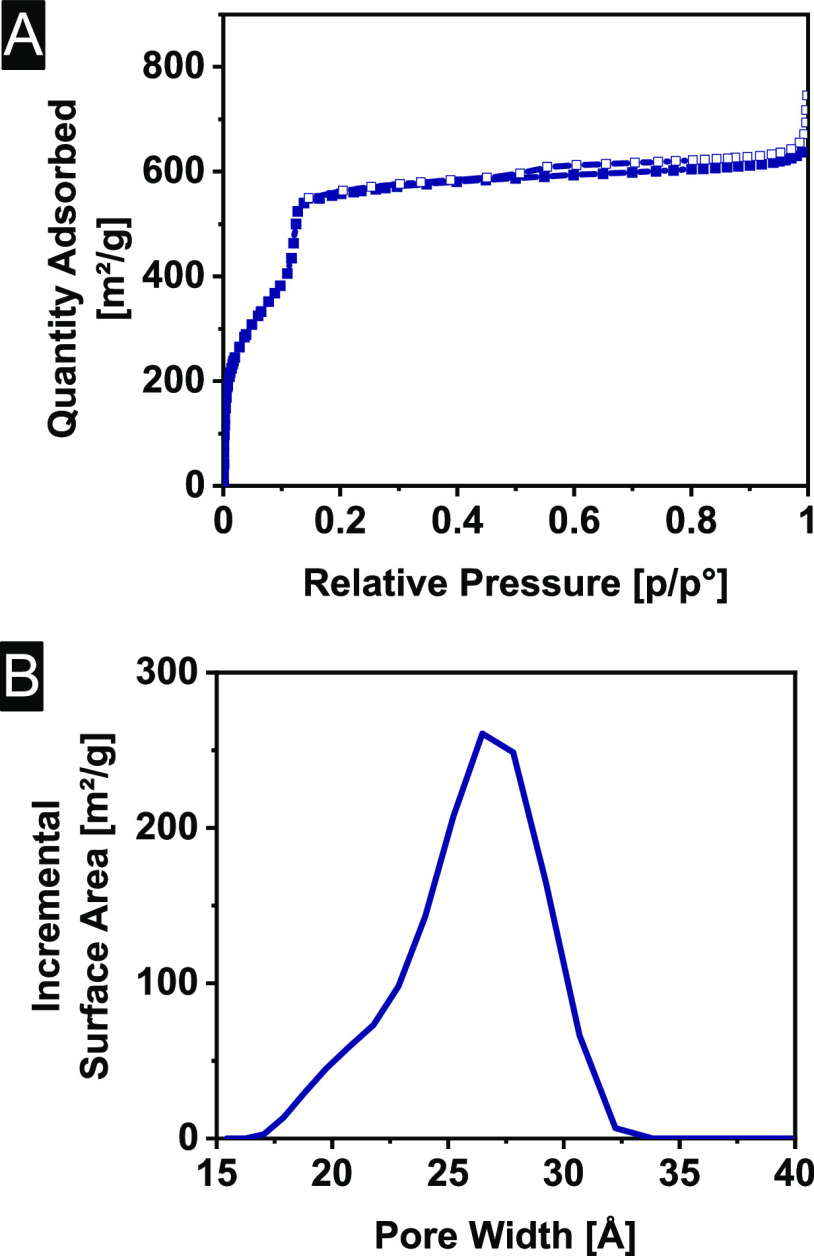
(A) N_2_ sorption isotherms of Me_3_TFB-(NH_2_)_2_BD. Filled symbols represent the adsorption branch; empty symbols represent the desorption branch and (B) their respective pore size distributions.

As a next step, N_2_ sorption measurements were carried out to determine the BET surface area. All three samples of Me_3_TFB-(NH_2_)_2_BD show almost identical adsorption–desorption isotherms, confirming the good repeatability of the synthesis (Figure 3A). The isotherm can be classified as a type IVb isotherm. The large uptake of N_2_ below *p*/*p*^0^ < 0.1 indicates the microporosity of the sample even if the pore size distribution—as determined by an HS-2D-NLDFT model—is slightly exceeding the definition of micropores with 2.7 nm (Figure 3B). The fit of the HS-2D-NLDFT model can be found in Figure S19. The average BET surface area was calculated using the Rouquerol criteria^67^ to apply the BET theory to microporous materials. On average a BET surface area of 1624 ± 89 m^2^/g was found (Figure S18). The pore volume at *p*/*p*^0^ = 0.95 was measured to be 0.90 ± 0.05 cm^3^/g.

**Figure 4:**
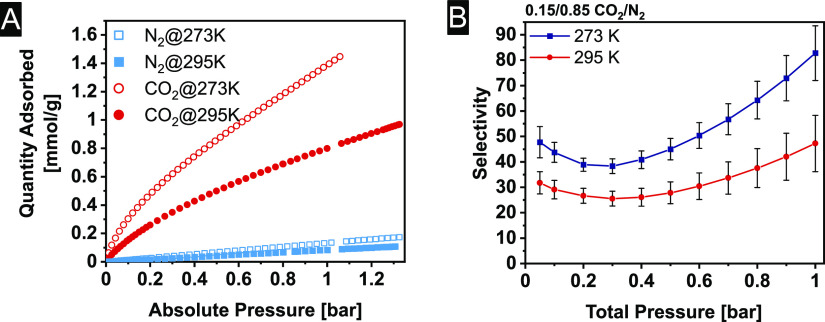
(A) Adsorption isotherms of CO_2_ and N_2_ at 273 and 295 K. (B) IAST selectivity between 0.05 and 1 bar of a 0.15/0.85 CO_2_/N_2_ mixture.

To study whether Me_3_TFB-(NH_2_)_2_BD can potentially be used in CO_2_ capture and storage, N_2_ and CO_2_ sorption experiments at 273 and 295 K have been conducted (Figure 4A). The underlying data for each of the three experiments that compose this data set can be found in the Supporting Information (Figure S20). The aromatic amine groups on the (NH_2_)_2_BD linker are expected to act as potential adsorption sides for CO_2_, which should lead to higher adsorption quantities compared to the Me_3_TFB-BD.^66^ The N_2_ adsorption isotherms of Me_3_TFB-(NH_2_)_2_BD are linear, and the CO_2_ isotherms show a significant increase at low pressures and resemble a Langmuir isotherm without reaching the plateau up to 1 bar. At 1 bar, CO_2_ adsorption capacities of 1.12 ± 0.26 mmol/g at 273 K and 0.72 ± 0.07 mmol/g at 295 K are found (Table 1). In comparison, Me_3_TFB-BD adsorbs 0.76 ± 0.03 mmol/g at 273 K and 0.41 ± 0.01 mmol CO_2_/g at 295 K at 1 bar.^66^ The incorporation of aromatic amine groups leads, therefore, to an increase in CO_2_ adsorption of 47 ± 13% at 273 K and 77 ± 10% at 295 K. In comparison to reported porous imine-based networks and COFs, which have adsorption capacities of 1.05–4.70 mmol/g at 273 K and 1 bar and 0.70–2.613 mmol/g at 298 K and 1 bar,^33,49,68,69^ Me_3_TFB-(NH_2_)_2_BD has a modest adsorption capacity. As explained in the Introduction, very recently, Yaghi and co-workers published COF-609, which contains aliphatic amine groups.^51^ They reported on approximately 2.1 mmol/g CO_2_ at 1 bar at 298 K. It is noted that the difference between COF-609 and Me_3_TFB-(NH_2_)_2_BD is not only the type of amine, i.e., aliphatic vs aromatic, but also the number of primary and secondary amine groups that can interact with CO_2_. COF-609 consists of two primary, two secondary, and one tertiary amine per repeating unit, while Me_3_TFB-(NH_2_)_2_BD contains only two aromatic amine groups per repeating unit, assuming a 100% conversion during linker exchange. These differences are believed to contribute to the differences in CO_2_ adsorption. Furthermore, Yaghi’s COF-609 contains additional triazine moieties that can contribute to CO_2_ adsorption,^50^ and their aliphatic amines form carbamates with CO_2_, leading to chemisorbed gas. Chemisorption interactions, which cover covalent bond formation, are energetically stronger than physisorption interactions, which are mainly based on Van der Waals interactions.^1^

**Table 1:** Quantities of N_2_ and CO_2_ Adsorption at Different Temperatures and Pressures and the Calculated IAST Selectivity Values

COF	pressure [bar]	temperature [K]	quantity adsorbed N_2_[mmol/g]	quantity adsorbed CO_2_[mmol/g]	IAST selectivity (CO_2_/N_2_ 0.15/0.85)
Me_3_TFB-(NH_2_)_2_BD	1 bar	273 K	0.12 ± 0.02	1.12 ± 0.26	83 ± 11
		295 K	0.07 ± 0.02	0.72 ± 0.07	47 ± 11
	150 mbar	273 K	0.016 ± 0.003	0.342 ± 0.046	
		295 K	0.009 ± 0.002	0.183 ± 0.025	
	4 mbar^b^	273 K	0.001 ± 0.000	0.022 ± 0.000	
		295 K	0.001 ± 0.000	0.007 ± 0.001	
Me_3_TFB-BD^a^	1 bar	273 K	0.10 ± 0.01	0.76 ± 0.03	7.7 ± 0.2
		295 K	0.05 ± 0.00	0.41 ± 0.01	7.4 ± 1.2

a Data used from previous published work to facilitate a comparison.^66^

b Data point closest to a partial pressure of CO_2_ (0.4 mbar) for DAC.

Moreover, the quantity of adsorbed N_2_ at 1 bar was measured to be 0.12 ± 0.02 mmol/g at 273 K and 0.07 ± 0.02 mmol/g at 295 K and thus much lower compared to CO_2_ adsorption. For direct air capture, CO_2_ adsorption at 0.04 mbar is relevant. Since the measured CO_2_ isotherm is not recorded at such low pressures, the first data point at approximately 4 mbar was used to evaluate the material for direct air capture applications. At 4 mbar, Me_3_TFB-(NH_2_)_2_BD adsorbed 0.0222 ± 0.0004 mmol/g CO_2_ at 273 K, which is less than that reported by Das et al. (∼0.3 mmol/g at 273 K and 4 mbar).^69^ Moreover, with 0.304 mmol/g at 273 K at an even lower partial pressure of 0.4 mbar, Yaghi and co-workers reported a higher adsorption capacity.^51^ This shows that the material is not suitable for direct air capture.

Therefore, we looked into the adsorption capacity at 150 mbar since this is relevant at coal flue gas conditions. At this pressure, the quantity of adsorbed CO_2_ was measured to be 0.342 ± 0.046 mmol/g at 273 K and 0.183 ± 0.025 mmol/g at 295 K, respectively (Table 1). The quantities of adsorbed N_2_ are 0.016 ± 0.003 mmol/g at 150 mbar and 273 K and 0.009 ± 0.002 mmol/g at 295 K and 150 mbar. In comparison with literature, the CO_2_ adsorption capacity at 150 mbar is lower than the capacity of already reported imine-based porous polymers (0.8–1.36 mmol/g at 273 K and 0.4–0.9 mmol/g at 295 K).^33,49,68,69^

To study the interaction involved in Me_3_TFB-(NH_2_)_2_BD, the reversibility of CO_2_ adsorption was investigated over five adsorption–desorption cycles (Figure S21). From the adsorption–desorption isotherm, it can be seen that the desorption occurred without hysteresis except for the first run, indicating the reversibility of the isotherm. It was also shown that the adsorption–desorption cycles at 273 K have a comparable adsorption (1.4 mmol/g vs 1.3 mmol/g) during the first adsorption cycle compared to the other four cycles, which further supports the reversibility of adsorption. The reversibility of the adsorption–desorption isotherm is also given at 295 K (Figure S22). To further study the COF–CO_2_ interaction, the COF was subjected to CO_2_ adsorption at 273 K. Without any additional treatment, the sample was then measured by FT-IR and ssNMR immediately afterward to probe the formation of carbamates, which would indicate chemisorption of CO_2_. Both the FT-IR spectrum (Figure S5) and the ssNMR spectrum (Figure S16) did not show any difference compared to those of the as-synthesized Me_3_TFB-(NH_2_)_2_BD, and no carbamate bands or signals could be observed. In comparison to Yaghi’s COF-609 where the aliphatic amines groups were found to chemisorb CO_2_, the adsorption–desorption cycles and the lack of carbamate signals in the FT-IR and NMR spectra of Me_3_TFB-(NH_2_)_2_BD after exposure to CO_2_ indicate that the CO_2_–COF interaction is based on physisorption and that the CO_2_ adsorption–desorption is reversible. In terms of applications, these results suggest that the material can be regenerated at relatively low cost in an energy-efficient way.

The isotherms were used to calculate the IAST selectivity with our recently developed GraphIAST^53^ software at coal flue gas conditions. First, the entire selectivity value range was scanned between 0.05 and 1.0 bar and mole fractions ranging from 0.05 to 0.95. The mole fraction of 0.15 CO_2_/N_2_ was used to investigate the CO_2_/N_2_ IAST selectivity for flue gas separation. IAST selectivity values of 83 ± 11 at 273 K and 47 ± 11 at 295 K were found in a mixture of 0.15/0.85 CO_2_/N_2_ at 1 bar (Table 1). These high selectivity values display that the approach taken in the design of Me_3_TFB-(NH_2_)_2_BD is promising for gas separation applications. Here, it is useful to compare our results with that of recent literature. Das et al. studied an imine-based network and determined IAST CO_2_/N_2_ selectivities of 211 at 273 K and 100 at 298 K.^69^ These values were validated by breakthrough experiments, leading to a selectivity of 125 at 298 K, which is even higher than predicted by IAST. The quantities of adsorbed CO_2_ are 3.3 mmol/g at 273 K, and 2.4 mmol/g at 298 K. Another porous imine-based network, studied by Popp et al., adsorbs 1.8 and 1.2 mmol/g CO_2_ at 273 and 298 K, respectively, with an IAST selectivity value of 31 at 298 K and 1 bar.^68^ Huang et al. synthesized an imine-linked porphyrin COF, of which the channel walls were post-synthetically functionalized with carboxyl groups to enhance selectivity.^33^ The CO_2_ adsorption was reported to be 3.95 and 1.73 mmol/g at 273 and 298 K, respectively, and the CO_2_/N_2_ selectivity of a 15% mixture of CO_2_/N_2_ at 298 K and 1 bar was 77, an enhancement of more than a factor of 9 compared to the unfunctionalized pristine COF. These examples show that channel–wall postfunctionalization^48,49^ can significantly enhance the CO_2_/N_2_ selectivity, while our current approach using post-functionalization via linker exchange can lead to comparably good results. The adsorbed quantities significantly increased from Me_3_TFB-BD to Me_3_TFB-(NH_2_)_2_BD, not only indicating the successful linker exchange but also showing that the aromatic amine groups of the (NH_2_)_2_BD linker interacts with CO_2_.

## Conclusions

A novel COF Me_3_TFB-(NH_2_)_2_BD, which is not accessible with classical direct condensation reactions, was synthesized by the linker-exchange strategy in a facile two-step synthesis. The linkage could unambiguously be assigned to imine bonds based on the chemical shift and CP build-up curves in ssNMR, and the crystallinity and porosity were confirmed by PXRD and physisorption experiments. Upon the exchange of Me_3_TFB-BD to Me_3_TFB-(NH_2_)_2_BD, a BET surface area of 1624 ± 89 m^2^/g was found, which shows that the linker exchange maintains most of the COF porosity.

Furthermore, Me_3_TFB-(NH_2_)_2_BD was studied for gas separation applications of CO_2_ and N_2_. We found modest CO_2_ adsorption quantities and high CO_2_/N_2_ selectivity values based on IAST calculations under coal flue gas conditions (83 ± 11 at 273 K/1 bar and 47 ± 11 at 295 K/1 bar). The enhanced CO_2_ uptake in comparison with the pristine Me_3_TFB-BD COF is caused by the aromatic amine groups of the COF linker. While aliphatic amine groups bind CO_2_ by chemisorption as shown by Yaghi and co-workers, the adsorption–desorption cycles of Me_3_TFB-(NH_2_)_2_BD have shown that the interaction of aromatic amine groups with CO_2_ are based on physisorption. As physisorption is mainly based on weak Van der Waals interactions, we therefore anticipate that the regeneration of Me_3_TFB-(NH_2_)_2_BD would require less energy, making it an interesting candidate for carbon capture.

## Supporting Information Available

Synthetic procedures, PXRD spectra (experimental, Pawley refined, and modeled), N_2_ sorption isotherms (273 and 295 K) and BET surface area plots and fits of the NLDFT model, ssNMRs, TGA (PDF)
CIF files of the modeled COF structures (CIF) (CIF) (CIF) (CIF)

## Supplementary Material

am2c17672_si_001.pdf

am2c17672_si_002.cif

am2c17672_si_003.cif

am2c17672_si_004.cif

am2c17672_si_005.cif
