# Supplementary material for: Aromatic Amine-Functionalized Covalent Organic Frameworks (COFs) for CO2/N2 Separation
Source: ACS Appl Mater Interfaces. 2023 Jan 17;15(4):5118–27. doi: 10.1021/acsami.2c17672 (PMC9906623; doi:10.1021/acsami.2c17672)
Supplement: Supplementary file 1 — am2c17672_si_001.pdf [file am2c17672_si_001.pdf]

SUPPORTING INFORMATION

Aromatic Amine-Functionalized Covalent Organic Frameworks (COFs) for CO_2/N_2 Separation

Ellen Dautzenberg[a], Guanna Li[a,b], Louis C. P. M. de Smet[a]*

[a] Laboratory of Organic Chemistry, Wageningen University, Stippeneng 4, 6708WE Wageningen, The Netherlands

[b] Biobased Chemistry and Technology, Wageningen University, Bornse Weilanden 9, 6708WG Wageningen, The Netherlands

Contact details: louis.desmet@wur.nl (Louis C.P.M. de Smet*, +31-317481268).

Author Contributions: L.C.P.M.d.S. and E.D. devised the project. E.D. planned and performed the experiments. G.L. performed the modelling. E.D. and L.C.P.M.d.S. analyzed the data. L.C.P.M.d.S. guided the project. E.D. wrote the first draft, and all authors gave input.

Contents

1. General Information....3

1.1 Materials....3

1.2 Instrumentation....3

1.3 Computation Details....4

2. Synthetic Procedures....5

3. Powder X-Ray Diffraction Analysis....6

4. ^{13}C CPMAS ssNMR Spectra....8

5. Nitrogen Sorption Analysis....12

6. CO2 and N2 adsorption at 273 K and 295 K....13

7. Thermogravimetric analysis....14

8. FT-IR spectra....14

9. Direct Condensation of $Me_3TFB-(NH_2)_2BD$....15

10. DFT modelling....17

11. References....18

1. Direct Condensation of $Me_3TFB-(NH_2)_2BD$

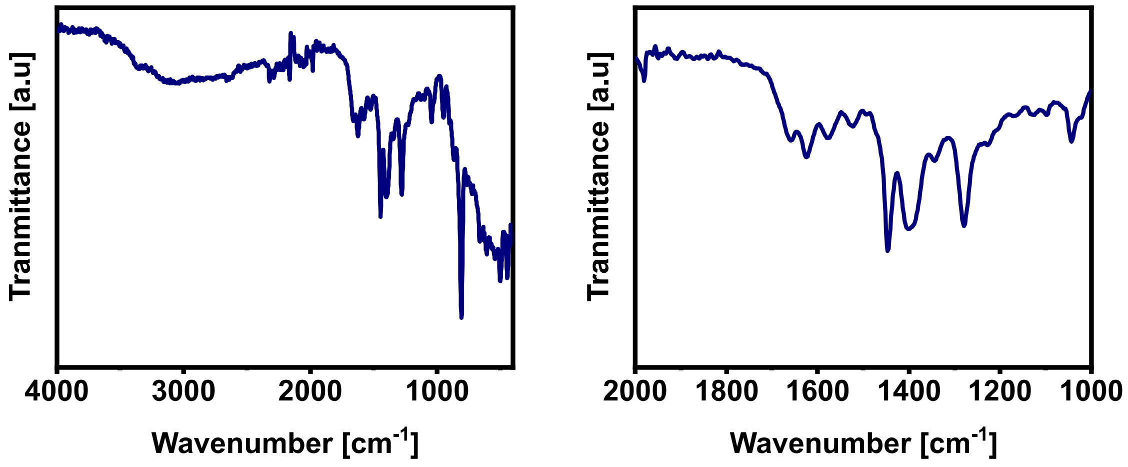

Figure S1. FT-IR spectrum of $Me_3TFB-(NH_2)_2BD$ (left) and region spectrum between 2000-1000 cm^{-1} (right).

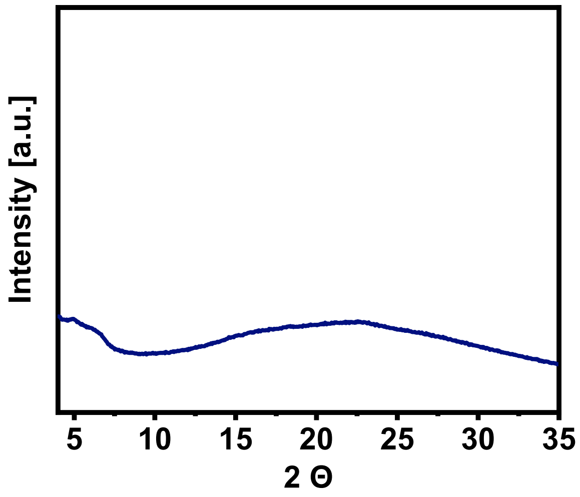

Figure S2. PXRD pattern after the direct condensation of $Me_3TFB-(NH_2)_2BD$.

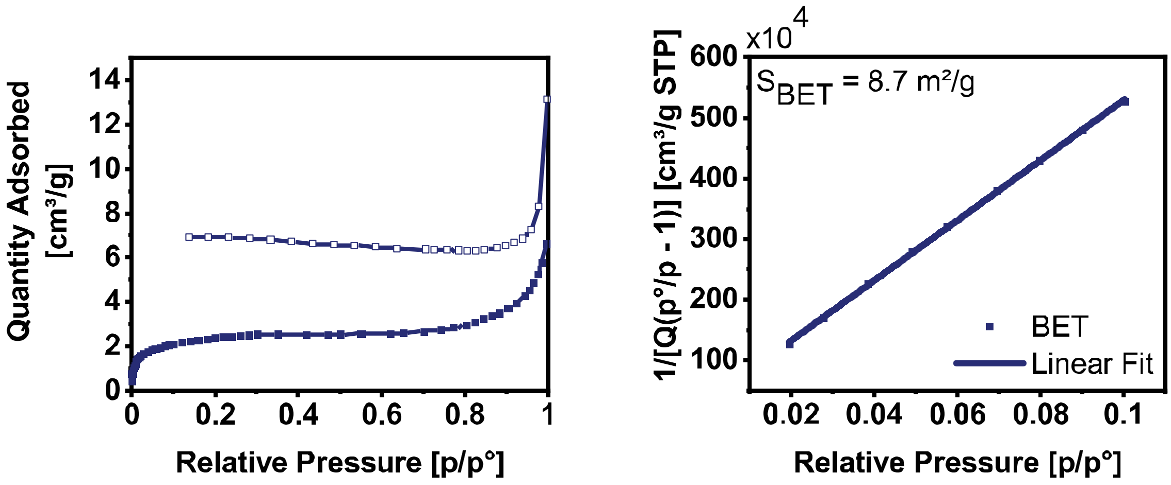

Figure S3. Adsorption (solid symbols)-desorption (open symbols) isotherm (left) and linear fit to calculate the BET surface area (right).

2. FT-IR

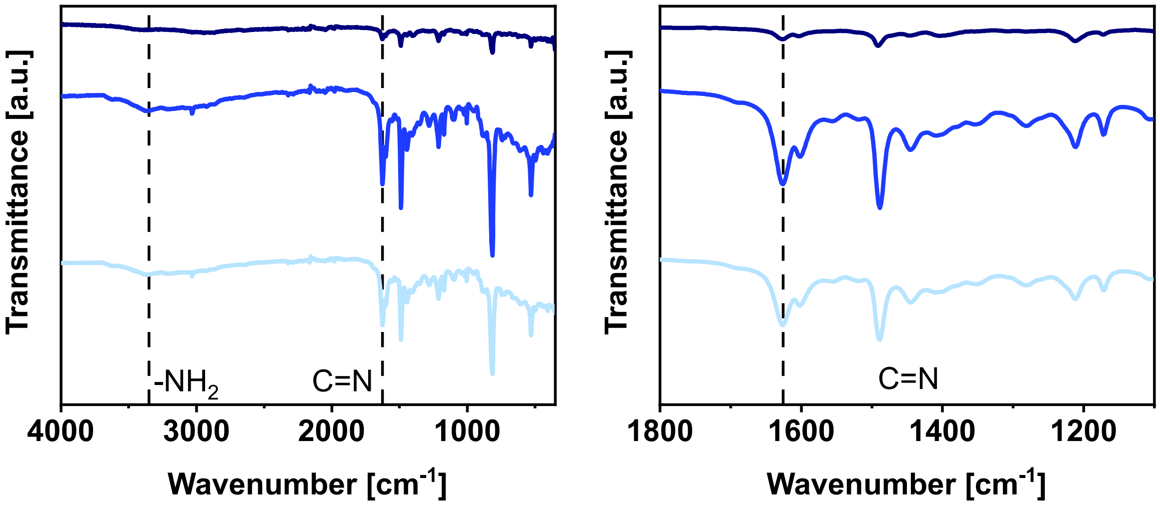

Figure S4. FT-IR spectra of the $Me_3TFB-(NH_2)_2BD$ COF triplicates (left) and zoom-in spectra in the relevant region for C=N imine stretch (right).

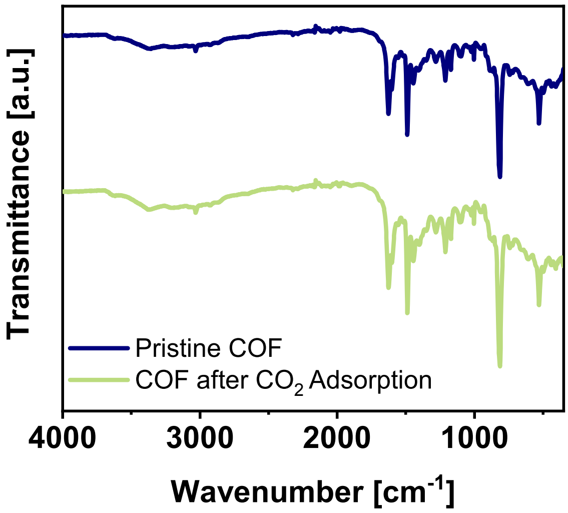

Figure S5. FT-IR spectra of the pristine COF and COF after a CO_2 adsorption cycle.

3. Powder X-Ray Diffraction Analysis

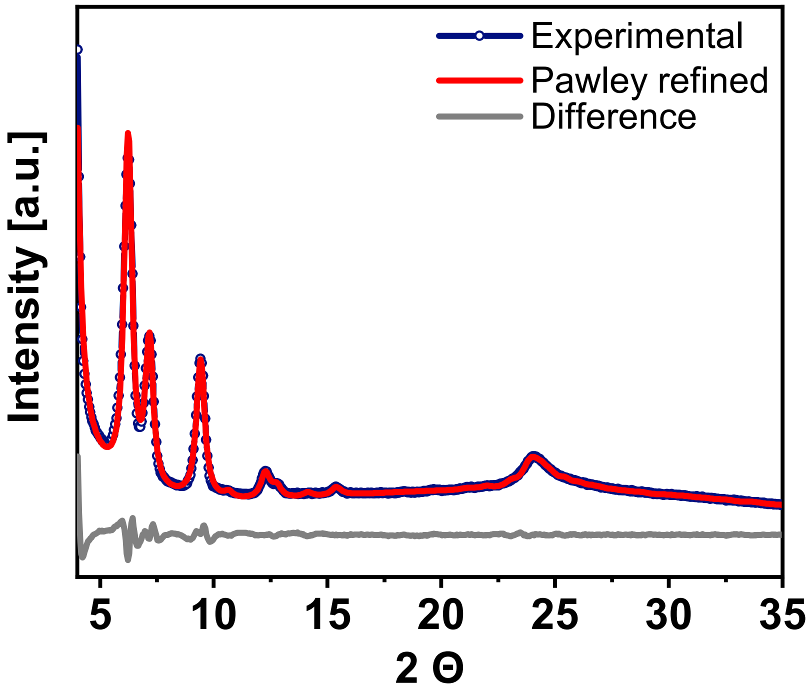

Figure S6. PXRD pattern of the second batch of $Me_3TFB-(NH_2)_2BD$, including Pawley refinement and the difference between the experimental and Pawley-refined pattern.

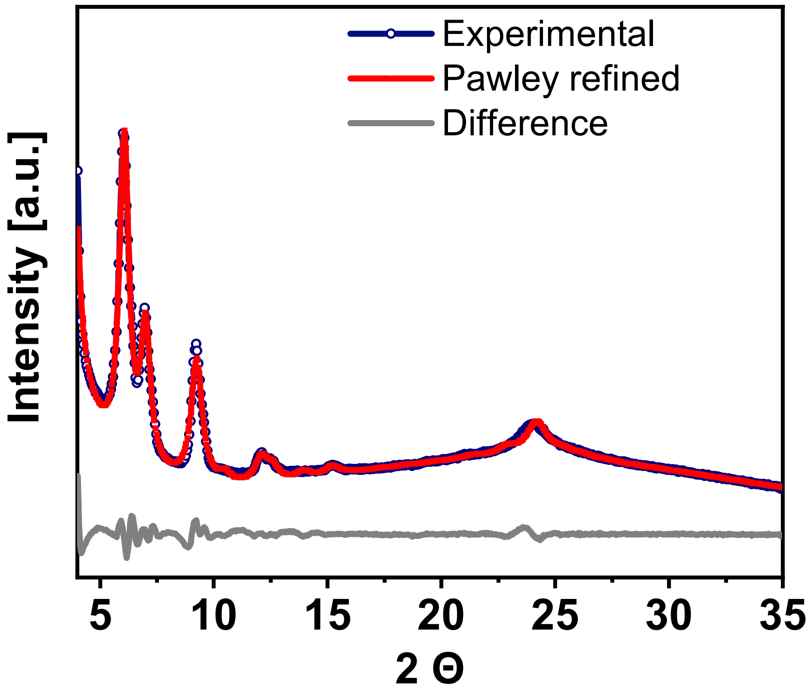

Figure S7. PXRD pattern of the third batch of $Me_3TFB-(NH_2)_2BD$, including Pawley refinement and the difference between the experimental and Pawley-refined pattern.

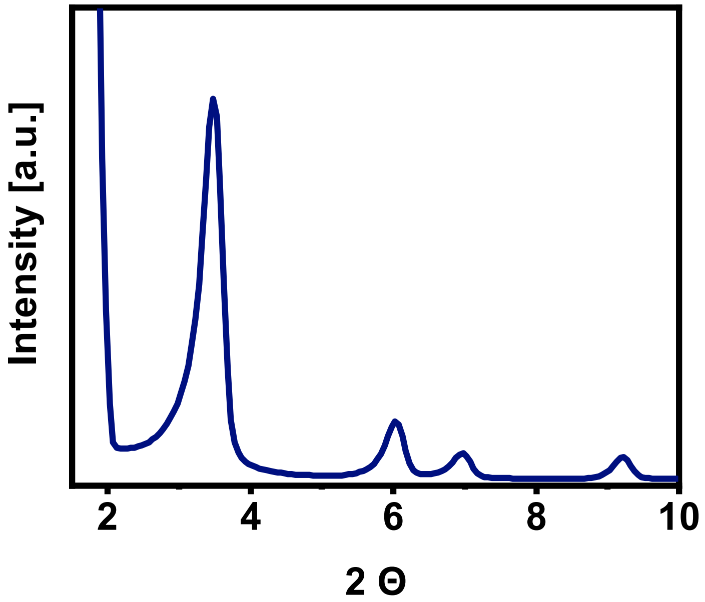

Figure S8. PXRD pattern from 1-10° to facilitate resolving the diffraction peak at 3.5°.

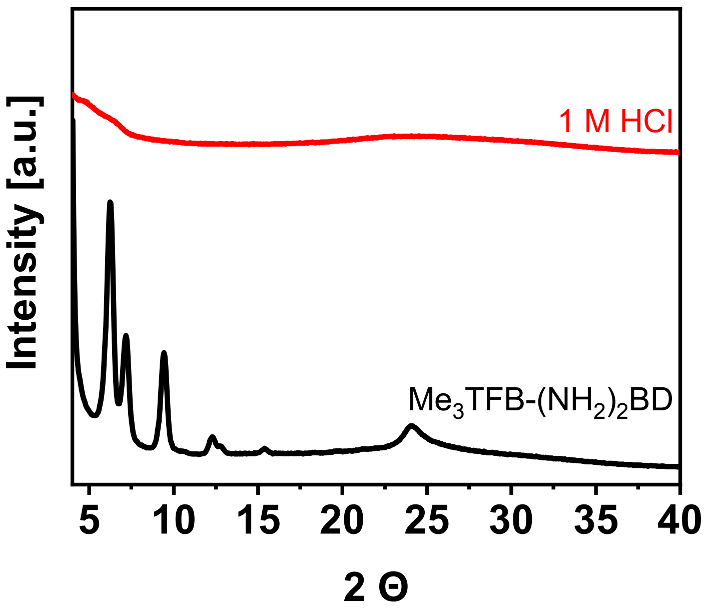

Figure S9. PXRD patterns of $Me_3TFB-(NH_2)_2BD$ as synthesized (black) and after exposure to acid (1 M HCl, 5 days), followed by re-isolation and drying at 120 °C overnight (red).

4. ^{13}C CPMAS ssNMR Spectra

$Me_3TFB-(NH_2)_2BD$

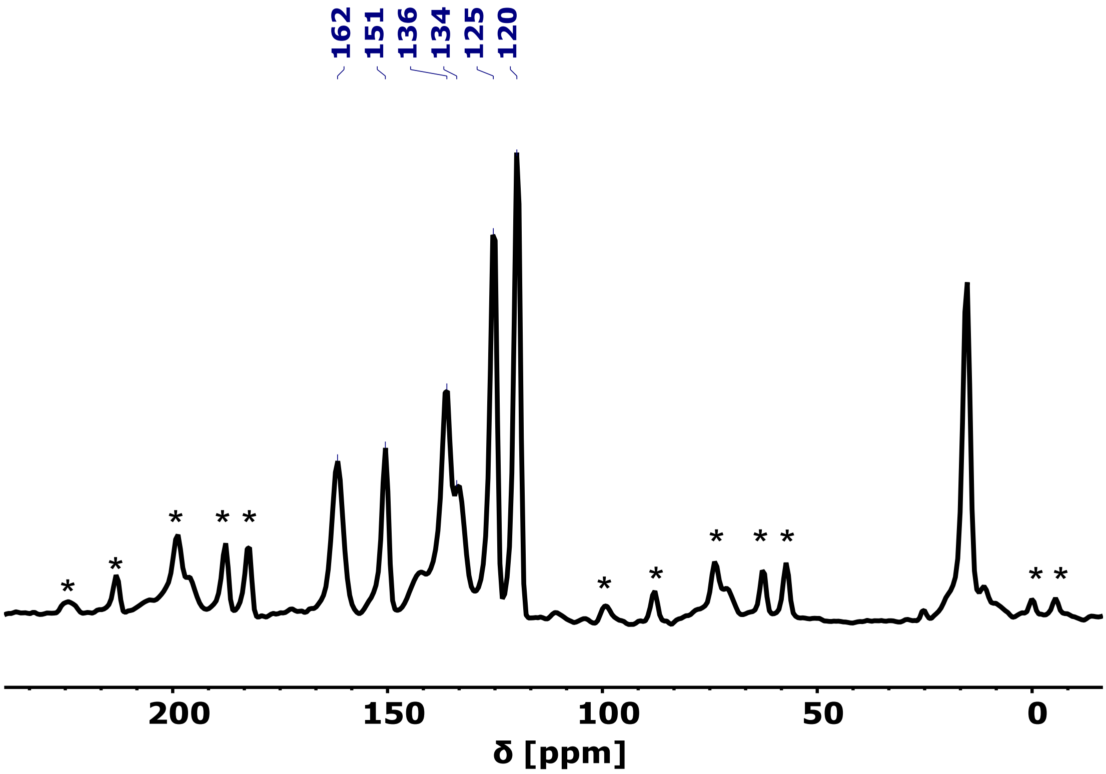

Figure S10. ^{13}C CPMAS solid-state NMR spectrum at 11 kHz. Spinning side bands, here indicated by an asterisk. The signal at 162 ppm can be assigned to the imine-bound carbon.

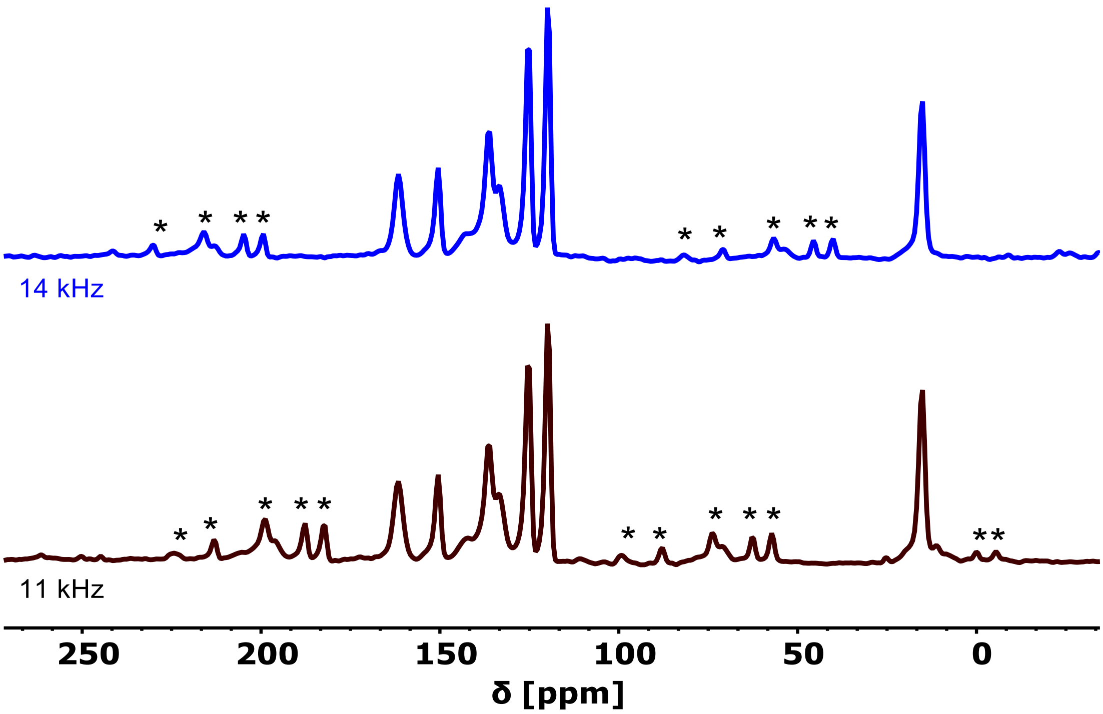

Figure S11. ^{13}C CPMAS solid-state NMR spectra at 14 kHz and 11 kHz to determine spinning side bands.

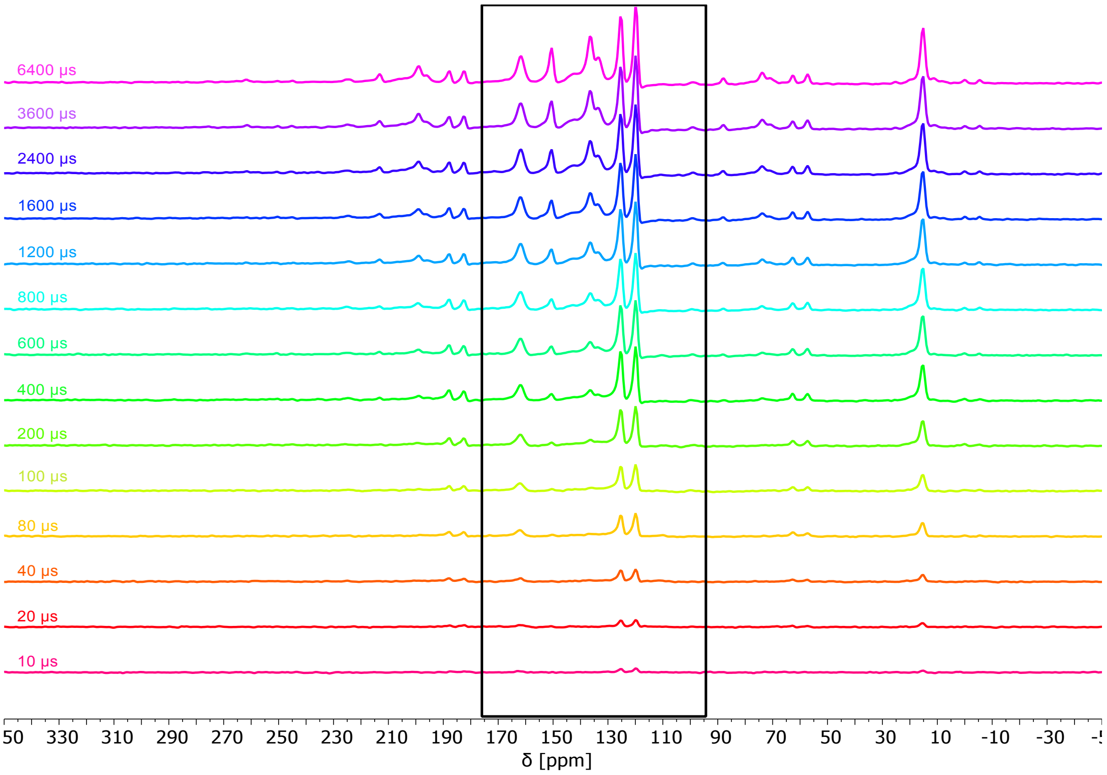

Figure S12. ^{13}C CPMAS solid-state NMR spectra of $Me_3TFB\text{-}(NH_2)_2BD$ at 11 kHz and different contact times.

2-Phenylbenzimidazole

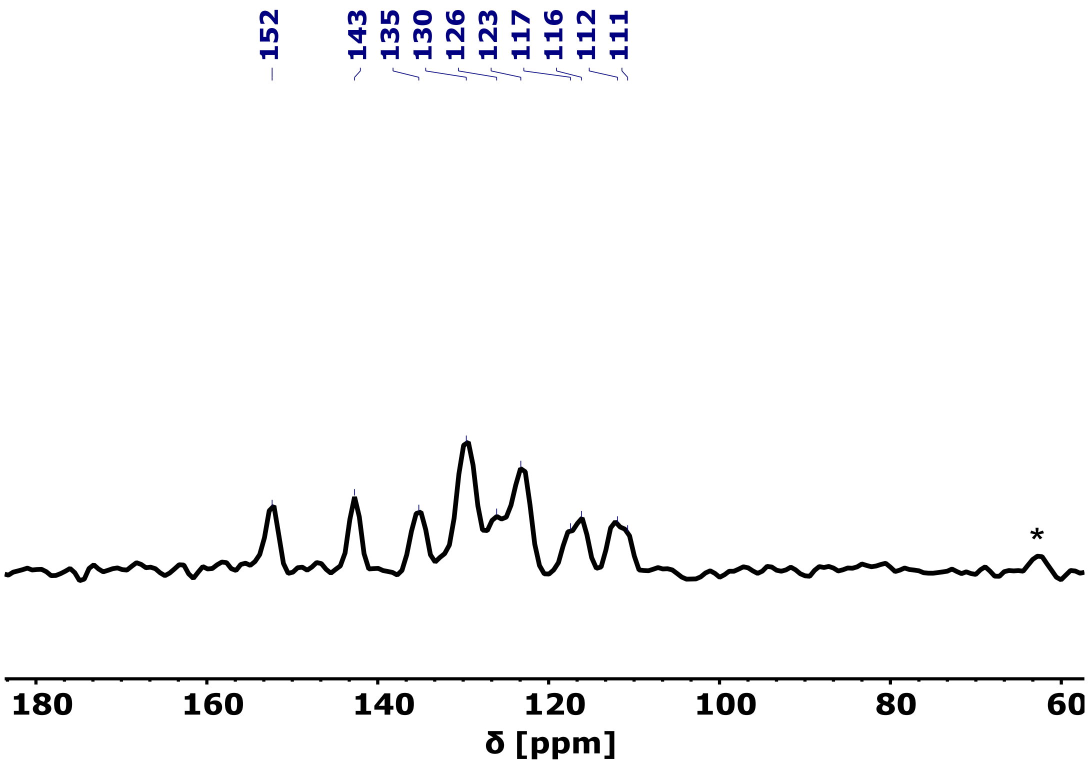

Figure S13. ^{13}C CPMAS solid-state NMR spectrum of 2-phenylbenzimidazole at 11 kHz. Spinning side bands, here indicated by an asterisk were determined by comparing different MAS frequencies. The signal at 152 ppm can be assigned to the benzimidazole-bound carbon.

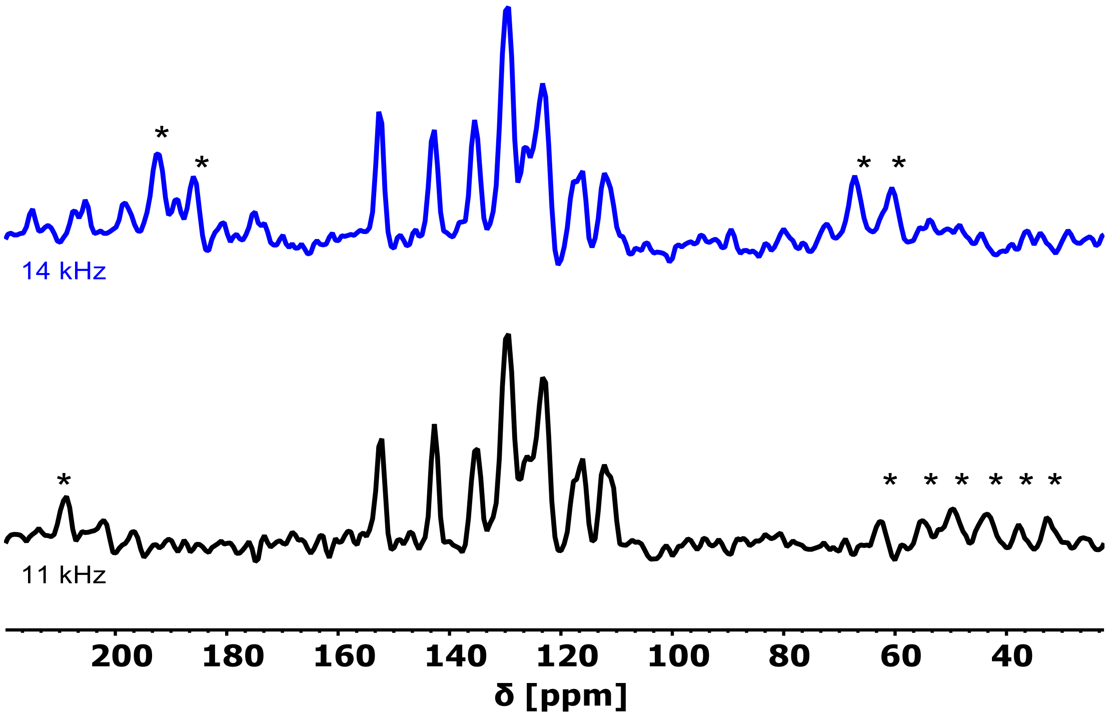

Figure S14. ^{13}C CPMAS solid-state NMR spectra of 2-phenylbenzimidazole at 14 kHz and 11 kHz to determine spinning side bands.

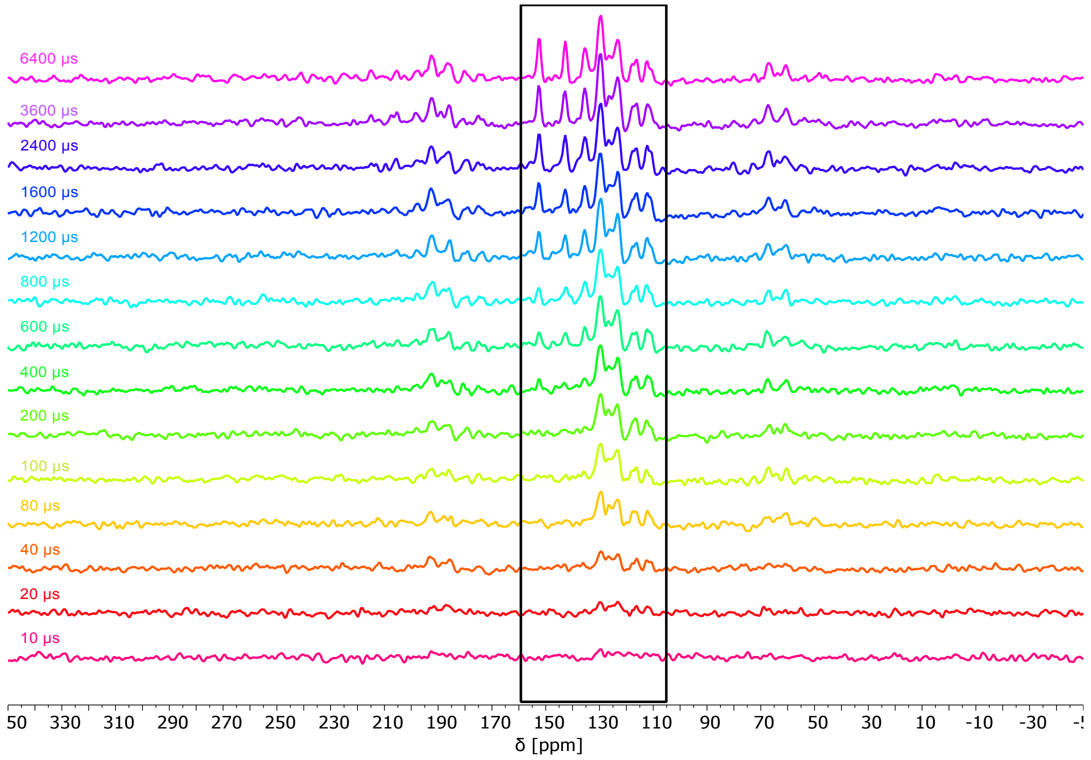

Figure S15. ^{13}C CPMAS solid-state NMR spectra of 2-phenylbenzimidazole at 11 kHz and different contact times.

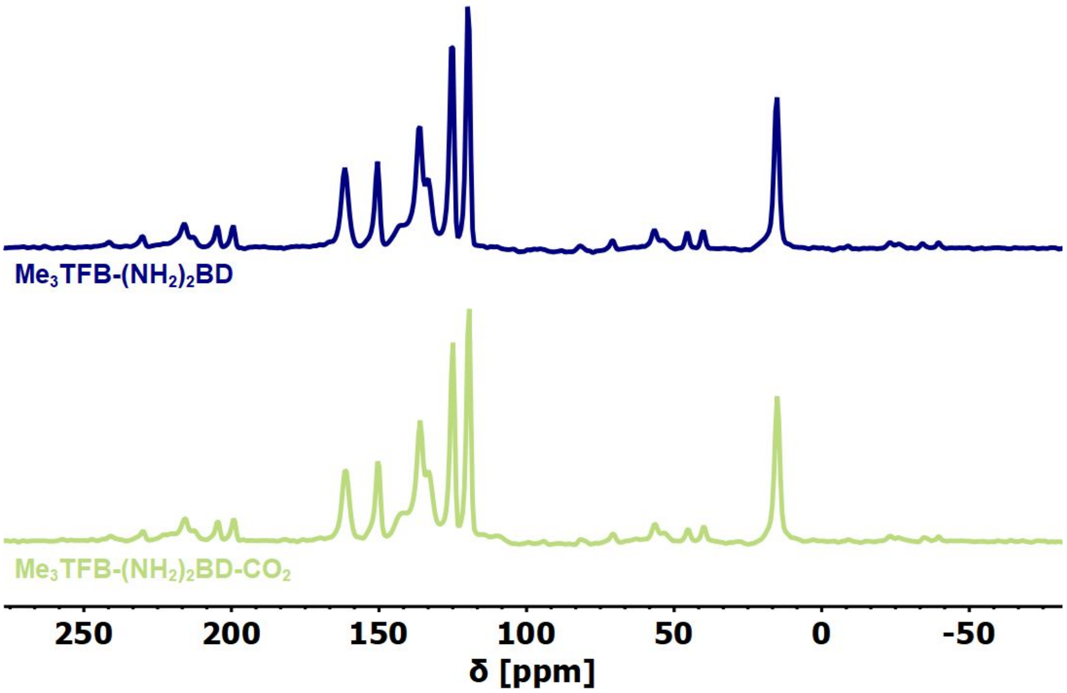

Figure S16. ^{13}C CPMAS solid-state NMR spectra of the pristine $Me_3TFB-(NH_2)_2BD$ COF and the COF spectrum after one CO_2 adsorption cycle. The identical spectra indicate the absence of carbamate moieties.

5. N_2 Sorption Analysis

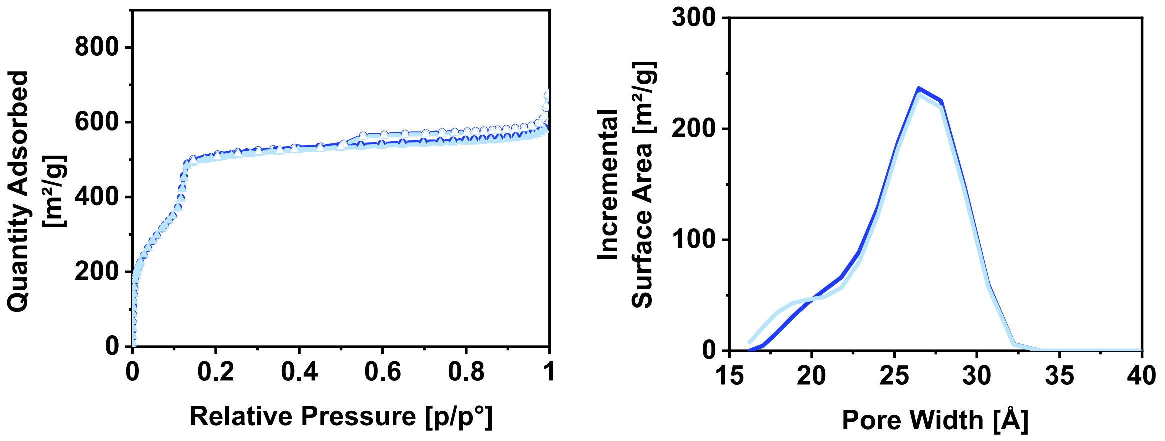

Figure S17. N_2 sorption isotherms of the $Me_3TFB\text{-}(NH_2)_2BD$ triplicates (left). Filled symbols represent the adsorption branch, empty symbol the desorption branch and their respective pore size distributions (right).

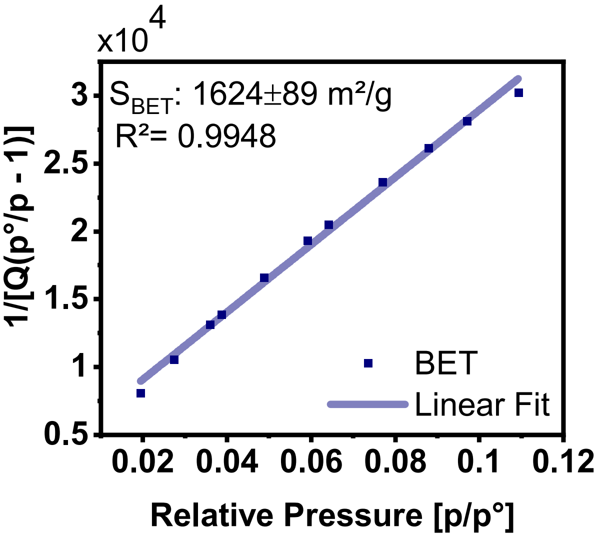

Figure S18. Linear fit to calculate the BET surface area, including R^2 and the average surface area of the triplicates including standard deviation.

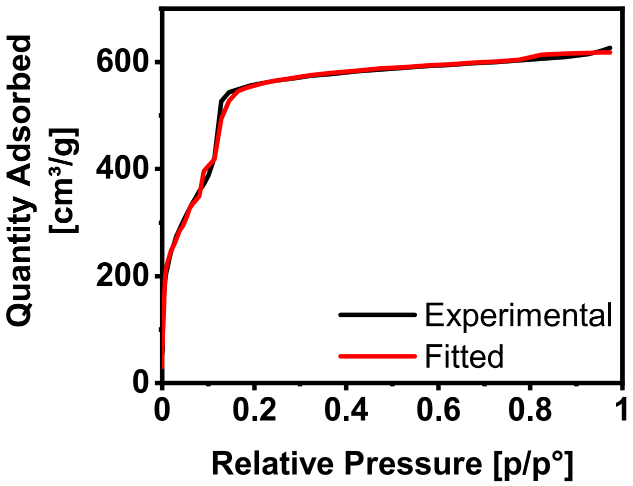

Figure S19. Experimental and theoretically modelled adsorption isotherm of $Me_3TFB-(NH_2)_2BD$.

6. CO_2 and N_2 Adsorption at 273 K and 295 K

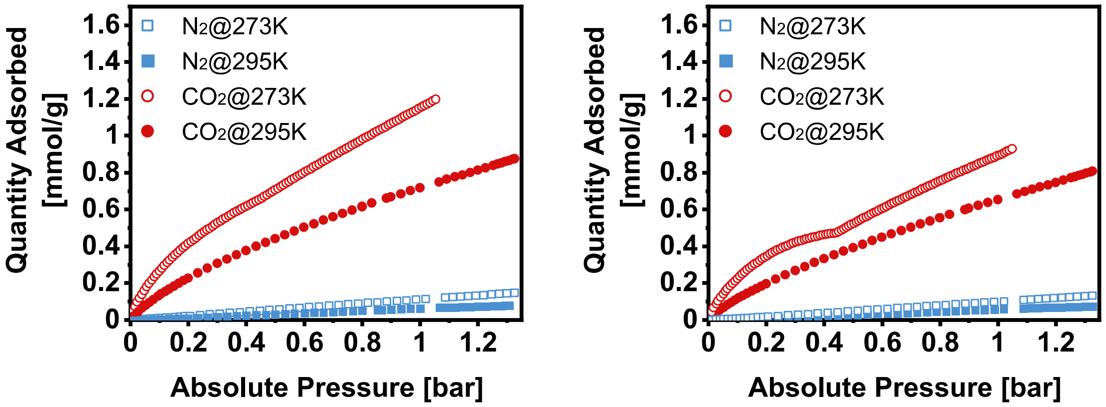

Figure S20. Nitrogen and carbon dioxide adsorption isotherms of the two other batches $Me_3TFB-(NH_2)_2BD$ at 273 K and 295 K.

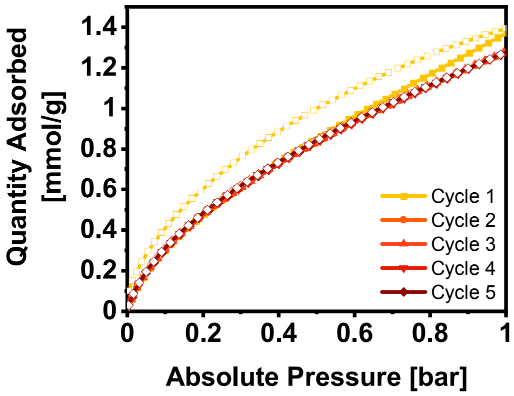

Figure S21 CO_2 adsorption (filled symbols) - desorption (open symbols) isotherms at 273 K over five cycles without degassing steps in between.

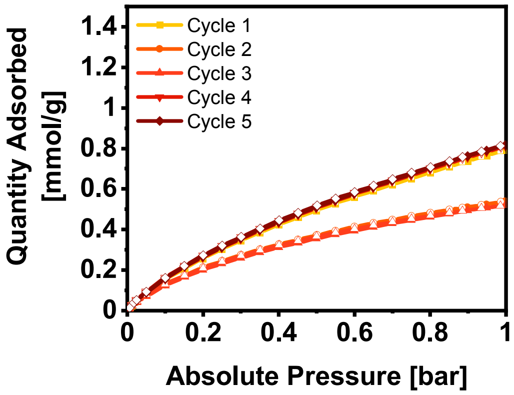

Figure S22 CO_2 adsorption (filled symbols) - desorption (open symbols) isotherms at 295 K over five cycles without degassing steps in between.

7. Thermogravimetric Analysis

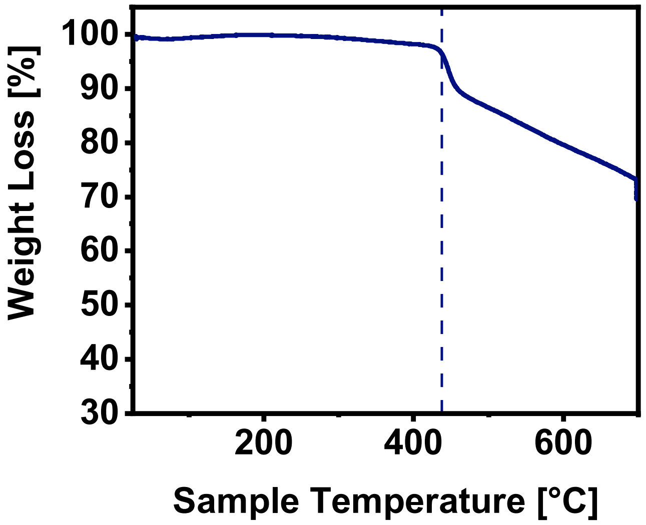

Figure S23. Thermogravimetric analysis of $Me_3TFB\text{-}(NH_2)_2BD$.

8. DFT Modelling

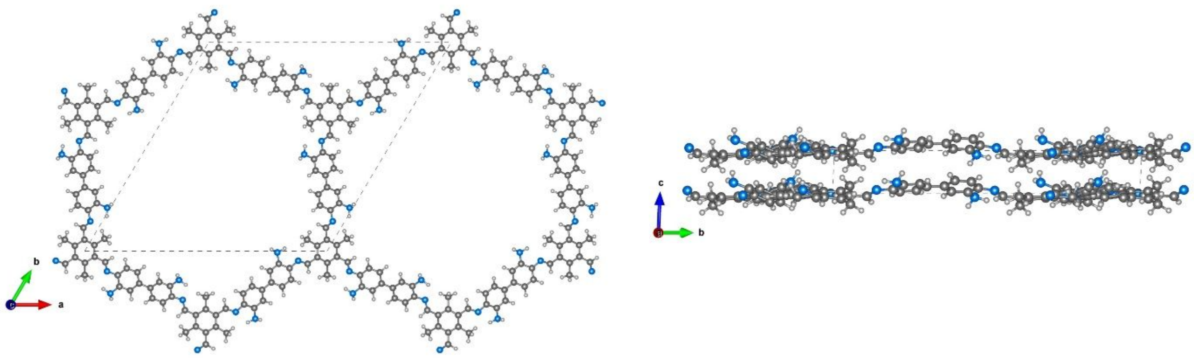

Figure S24. DFT-optimized crystal structure of $Me_3TFB\text{-}(NH_2)_2BD$ from top view (left) and side view (right). Coordinates available as separate files.
